# Metformin induces diarrhea in mice under over-eating conditions

**DOI:** 10.1007/s13340-025-00822-0

**Published:** 2025-06-05

**Authors:** Kotomi Chikama, Hiroshi Takemroi, Momoka Mizoguchi, Saho Furukawa, Koutarou Terada, Masafumi Ito, Hirotsugu Hirano, Takanori Miura, Koichi Doi, Megumi Horiya, Takehiro Kato, Daisuke Yabe, Takashi Shibata

**Affiliations:** 1 Department of Life Science and Chemistry, Graduate School of Natural Science and Technology, Gifu University, Yanagido 1-1, Gifu, 501-1193 Japan; 2 Taiko Pharmaceutical Co., Ltd., Honnmachi 1-4-1, Nishiku, Osaka 550-0005 Japan; 3 Department of Diabetes, Endocrinology and Metabolism/Department of Rheumatology and Clinical Immunology, Yanagido 1-1, Gifu, 501-1194 Japan; 4 Department of Diabetes, Endocrinology and Nutrition, Kyoto University Graduate School of Medicine, 54 Shogoin-Kawahara-cho, Sakyo-ku, Kyoto, 606-8507 Japan

**Keywords:** Metformin, Diarrhea, GLP-1, Cellulose, cAMP

## Abstract

Metformin, an oral medication for type 2 diabetes, causes severe diarrhea in approximately 5% of individuals with diabetes in Japan, leading them to discontinue treatment despite the drug efficacy, safety, and low economic burden. Given the absence of animal models for diarrhea, we previously proposed a mouse model for metformin-induced diarrhea using diabetic obese *db/db* mice. The diarrhea model exhibited elevated gene expression of glucagon-like peptide-1 (GLP-1), which was followed by increased expression of the Cl⁻ channel CFTR. However, it remains unclear which specific risk factors in the *db/db* mouse model are associated with the development of diarrhea. In this study, healthy C57BL/6 J mouse models with dietary modifications were used to replace *db/db* mice. Unexpectedly, C57BL/6 J mice fed diets containing 10% cellulose consumed more feed and gained weight more rapidly. Overnight fasting led to increased food intake once feeding resumed. The combination of these feeding conditions and metformin administration resulted in increased water content in their feces. Furthermore, the enhanced expression of GLP-1 and CFTR, the decrease in the abundance of the gut microbial *Firmicutes* family, and the alleviation of diarrhea symptoms by wood creosote share similarities with metformin-induced diarrhea in *db/db* mice. Although the administration of the GLP-1 receptor agonist Exendin-4 did not induce diarrhea in mice without metformin treatment, the GLP-1 receptor antagonist Exendin-3 (9–39) inhibited the development of diarrhea in mice treated with metformin. These results suggest that overeating, coupled with abnormal regulation of GLP-1 signaling, may be associated with an increased risk of metformin-induced diarrhea in mice.

**Supplementary Information:**

The online version contains supplementary material available at 10.1007/s13340-025-00822-0.

## Introduction

Metformin, a medication for type 2 diabetes mellitus, is the most frequently prescribed medication worldwide [1]. The proposed mechanism by which metformin ameliorates the symptoms of type 2 diabetes mellitus involves energy leakage in mitochondrial electron transport systems, which activates AMP-activated kinase (AMPK) [2], leading to inhibition of gluconeogenesis, stimulation glucose consumption, and improvement of insulin resistance. In addition, metformin has been found to increase the expression of glucagon-like peptide-1 (GLP-1) in the gut (ileum and colon) [3], which stimulates insulin secretion in pancreatic β-cells [4].

Despite the well-established clinical benefits of metformin, its adverse effects are significant, particularly in Japanese individuals with diabetes. Diarrhea is a serious adverse effect of metformin, reported in approximately 25–30% of individuals with diabetes prescribed this medication [5, 6]. Moreover, 5% of the individuals discontinue treatment due to severe symptoms. Several risk factors for metformin-induced diarrhea have been identified, including dosage, age, sex, body mass index, and liver and biliary disorders [6]. However, the relationship between metformin-induced diarrhea and lifestyle habits, such as the amount and rate of food consumption and food composition, has not been elucidated.

Recently, we established a mouse model of metformin-induced diarrhea using diabetic obese *db/db* mice [7, 8], which share several risk factors (body mass index and liver and biliary disorders) with individuals with diabetes. Since *db/db* mice lack the receptor for leptin, which is secreted from adipocytes after a meal and suppresses appetite in the feeding center of the hypothalamus, these mice exhibit an overeating phenotype [9]. GLP-1 also exerts appetite-suppressive actions, and its analogs are approved as weight-loss medications [10].

Albeit with a small incidence, GLP-1 induces diarrhea [11–13] and we have shown that excessive secretion of GLP-1 in metformin treated *db/db* mice may be a cause of diarrhea. When GLP-1 binds to its receptor [8], which is coupled with the Gq protein α subunit, the receptor complex recruits adenylyl cyclase and triggers intracellular cyclic AMP (cAMP) signaling. Then, cAMP activates protein kinase A (PKA), which induces cAMP-dependent transcription via the transcription factor cAMP response element binding protein (CREB) [8, 14]. This signaling pathway up-regulates the expression of several genes, including those regulating osmolality and water uptake, such as the cystic fibrosis transmembrane conductance regulator (CFTR: a chloride (Cl^−^) channel) and aquaporin (AQP: a water channel) [15]. The cholera toxins and bacterial enterotoxins are examples of cAMP-mediated diarrhea caused by the dysregulation of water absorption [16]. The Na^+^/H^+^ exchanger NHE3 is also regulated by GLP-1 [17, 18] and involved in metformin-induced diarrhea in *db/db* mice [19].

Here, we demonstrate that metformin increases the risk of diarrhea even in healthy C57BL/6 J mice under abnormal feeding conditions, as represented by the enhanced rate of food intake. A high-cellulose diet induces overeating and weight gain in mice. Additionally, metformin administration in conjunction with refeeding after fasting increases fecal water content, resulting in diarrhea-like symptoms. The metformin-induced diarrhea observed in the present overeating model shared patterns of mRNA expression and microbial composition with those in *db/db* mice. These results suggest that overeating may trigger metformin-induced diarrhea in mice.

## Materials and methods

### Mouse experiments

All animal experimental procedures were approved by the Animal Committee in Gifu University (2019-192, 2021-155, and AG-P-N-20240176). C57BL/6 J mice (6 W male) were purchased from SLC Japan (Shizuoka, Japan). The animals were maintained under a 12-h light/dark cycle (08:00–20:00) and received a standard rodent diet CE-2 or 10% cellulose containing CE-2 (CLEA Japan, Inc., Tokyo, Japan). High fat (45 kcal%)/high sucrose (25 kcal%) (HF/HS) diet (D22455) was purchased from Research Diet, Inc. (New Brunswick, NJ, USA).

Metformin hydrochloride (COMBI-BLOCKS, San Diego, CA) and wood creosote [20] were suspended in 0.5% Tween 80 (Kanto Kagaku, Tokyo, Japan). The drugs were orally administrated to the mice twice daily at 19:00–20:00 and 8:00–9:00. Exendin-3 (9–39) amide and Exendin-4, purchased from PEPTIDE (Osaka, Japan) and MedChemExpress (NJ, USA), respectively, were suspended in phosphate-buffered saline (PBS). A dose of 1 μg/100 μL PBS per mouse of these peptides was administered via intraperitoneal injection (I.P.). Subsequently, metformin was administered orally (P.O.). The dose of GLP-1 receptor agonists/antagonists was determined based on previous reports [21, 22].

Body weight was measured and the feces samples were collected in the morning. Feces from eight mice were randomly collected into 2 mL sample tubes and weighed. The samples were dehydrated overnight and the weight was measured again.

Glucose tolerance assay was performed by orally treatment with 1 g glucose after 3 h fasting, and blood glucose was measured by the Glucocard (Arkray Co. Ltd., Kyoto, Japan). Mouse total GLP-1 ELISA kit (YK161) was purchased from Yanaihara Institute Inc. (Shizuoka, Japan). Blood was collected via the tail vein from non-metformin-treated mice between 8:00 and 9:00 a.m. to measure basal GLP-1 levels. To evaluate the effects of metformin, blood was collected from the heart prior to colon recovery.

### Total bile acids measurement

The mucosa fractions were suspended in five times volumes of phosphate-buffered saline (PBS) and centrifuged (12,000 g, 2 min); the supernatant was used to quantify total bile acids. The specific amount was normalized by protein concentration using the bicinchoninic acid protein assay method with bovine serum albumin as a standard. The deproteinization was performed as follows: 300 μL of the sample in PBS was mixed with 700 μL of acetonitrile (Kanto Chemical, Tokyo, Japan) and stirred for 1 min. The protein was then removed by centrifugation (12,000 g for 5 min). The supernatant was dried by evaporation at 80 °C and precipitates were resuspended in 100 μL of PBS.

Total bile acid levels were measured using the Total Bile Acids Assay Kit (Diazyme Laboratories, Inc., Poway, CA, USA). 68.5 µL of Reagent 1 were plated in a 96-well plate, to which 1 μL of the deproteinization sample, the diluted bile acid standard, and PBS (as blank) were added and the plate was incubated at 37 °C for 3 min. Then, 22.5 µL of Reagent 2 were added and the absorbance was immediately measured at 450 nm and denoted as A0. Each 1 min later, the absorbance at 450 nm denoted as A1〜10. Total bile acid levels were determined using the following equation: Sample (μM) = [Sample ΔA450/min—Blank ΔA450/min] / [Standard ΔA450/min—Blank ΔA450/min] × Standard (100 μM).

### Quantification polymerase chain reaction (qPCR) and microarray analyses

cDNA was synthesized from 1 µg total RNA for mice using 40 units of RNase inhibitor (Toyobo, Osaka, Japan), 25 pmol of random primer, 4 µL of 5 × RT buffer, 2 µL of 10 mM deoxynucleotides, and 100 units of ReverTraAce (Toyobo, Osaka, Japan) by incubating at 30 °C for 10 min and 42 °C for 1 h, followed by denaturation at 99 °C for 5 min. The cDNA was amplified in a reaction mixture (20 µL) containing Thunderbird SYBR qPCR Mix (Toyobo) and 3 pmol of each forward and reverse primer using MyiQ (Bio-Rad, Hercules, CA, USA). qPCR analysis was carried out under the following conditions: initial denaturation at 95 °C for 1 min, followed by 40 cycles of denaturation at 95 °C for 20 s, annealing at 58 °C for 30 s, and a final extension at 72 °C for 34 s. mRNA levels were expressed relative to glyceraldehyde 3-phosphate dehydrogenase (*Gapdh*) mRNA. The primers used are shown in the Supplementary Table.

### Statistical analysis

For all experiments, the data are expressed as the mean ± standard deviation (S.D.). One-way analysis of variance (ANOVA) and Student’s t-test were performed using the data analysis tool in Microsoft Excel (WA, USA) for statistical analysis. * and ** indicate *p* < *0.05* and *p* < *0.01*, respectively.

## Results

### Abnormal eating behavior is a risk factor of metformin-induced diarrhea in mice

In our previous studies, metformin-induced diarrhea was observed only in obese diabetic mice (*db/db*) when used as model subjects, and the metformin dose was set at 500 mg/kg/bid (1000 mg/kg/day) [8]. In addition, the administration of a single doses ≥ 900 mg/kg/day has been reported to cause moribundity, mortality, and clinical signs of toxicity in rats [23]. Therefore, in this study, we utilized healthy C57BL/6 J mice, administered metformin at a dose of 500 mg/kg/bid, and concentrated on feeding patterns. To reproduce the eating habits of obese peoples, C57BL/6 J mice (healthy mice) were initially fed a high-fat/high-sucrose diet for three weeks (Fig. S1A). The mice which were fed a high-fat, high-sucrose diet consumed slightly less food per day, but their body weight gain tended to increase (Fig. S1B and C).

Metformin treatment (500 mg/kg/bid) did not induce diarrhea, and the fecal moisture was slightly reduced in the mice of the high-fat/high-sucrose group (Fig. S1D and E). Although we expected that reduced food intake was due to increased levels of total serum GLP-1, the levels were reduced and not recovered by the metformin treatment (Fig. S1F). These results suggest that high-fat/high-sucrose diet feeding cannot not be replaced a model for metformin-induced diarrhea as observed in *db/db*. Interestingly, the atrophy of the colon was observed in mice feeding the high-fat/high-sucrose diet (Fig. S1D and G).

**Fig. 1:**
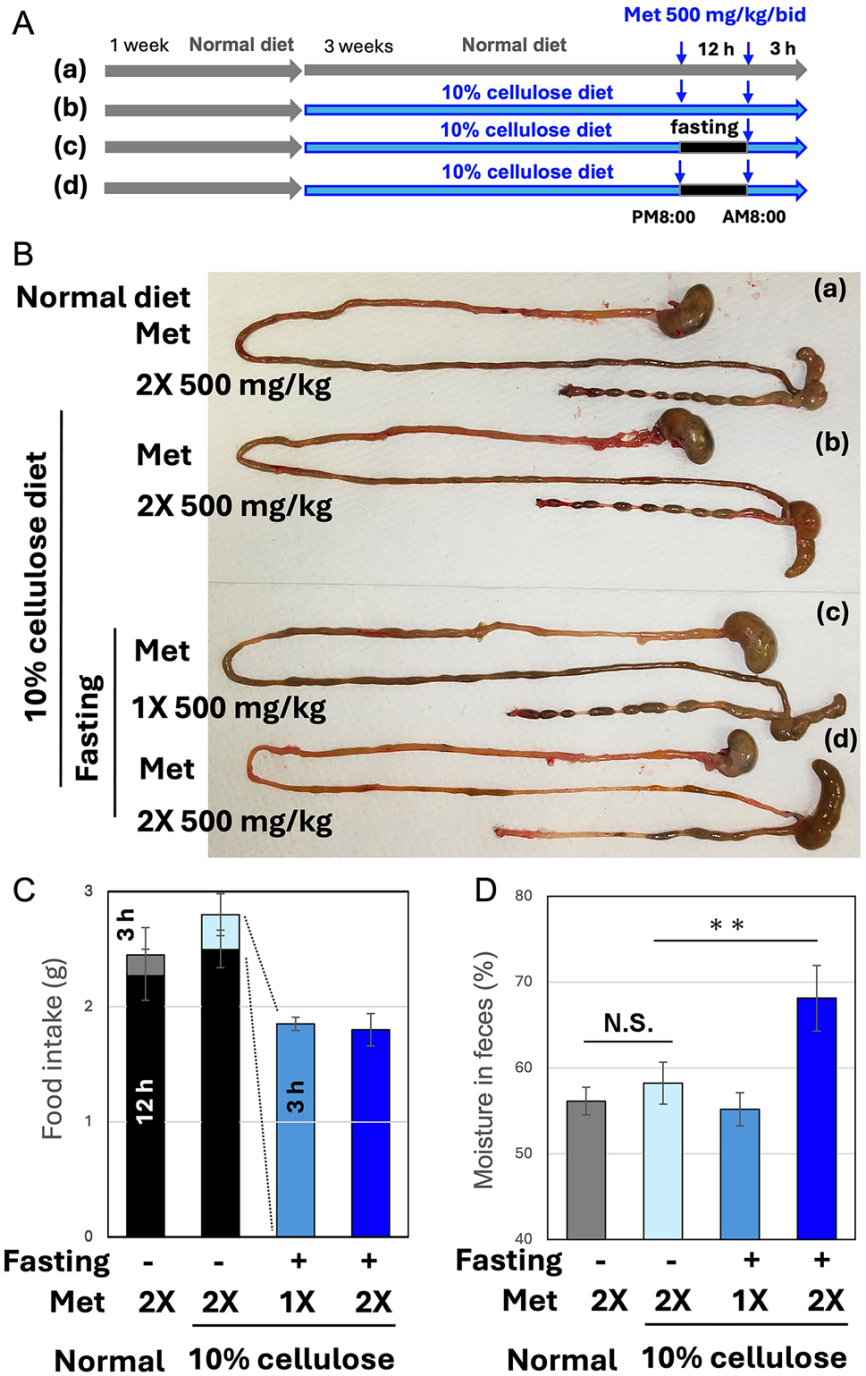
Overeating increases fecal water content after metformin treatment **A** Six-week-old male C57BL/6 J mice (*n* = *8* each) were fed a normal diet for 1 week (gray arrow). Subsequently (from seven-week-old), one group (**a**) continued on the normal diet, while the other groups (**b–d**) were switched to a 10% cellulose diet (blue arrow) for three weeks. On the night prior to the final day, metformin (Met: 500 mg/kg) was orally administered, except for group (**c**), which did not receive the administration. Groups (c and d) on the 10% cellulose diet underwent a 12-h fasting period (gray bar). Finally, all mice received metformin administration (Met: 500 mg/kg) and were fed without restriction. Images of the mice gut (**B**) are shown. **C**. Food consumption of the final 15 h (12 h during the night: black) and 3 h in the morning was monitored. Means and S.D. are shown. **D**. Water content in mice feces is shown. ** *p* < *0.01*

Next, instead of a high-fat/high-sucrose diet, the mice were fed a low-calorie diet containing 10% cellulose. In preliminary tests, (a) and (b) in Fig. 1A and B, although no significant differences in gut appearance were observed, some mice in group (b) excreted feces that were difficult to handle with forceps. Therefore, we examined the differences in feeding patterns, specifically focusing on whether the mice ingested food all at once by setting a fasting period for 12 h in night (c) and (d). Although the total food intake during the night (12 h) and in the morning (3 h) was lower in the fasting groups (c and d) compared to the no-restriction group (b), the fasting groups showed a higher intake during the refeeding period in the morning (3 h) (Fig. 1C). The administration of metformin to mice fed a 10% cellulose diet and subjected to fasting resulted in high fecal moisture (Fig. 1D). When mice were not subjected to fasting, fecal moisture did not change (around 55%), regardless of whether they were fed a normal diet or a 10% cellulose diet.

To determine whether diarrhea-like feces were caused by the amount of diet consumed by mice following the second administration of metformin, a restricted diet (0.5 g/mouse: refer from Fig. 1C) was provided (Fig. S2A). The dietary restriction improved fecal firmness and reduced water content, which was accompanied by a decrease in fecal size (Fig. S2B and S2C).

### Overeating is a risk factor for metformin-induced diarrhea in mice

**Fig. 2:**
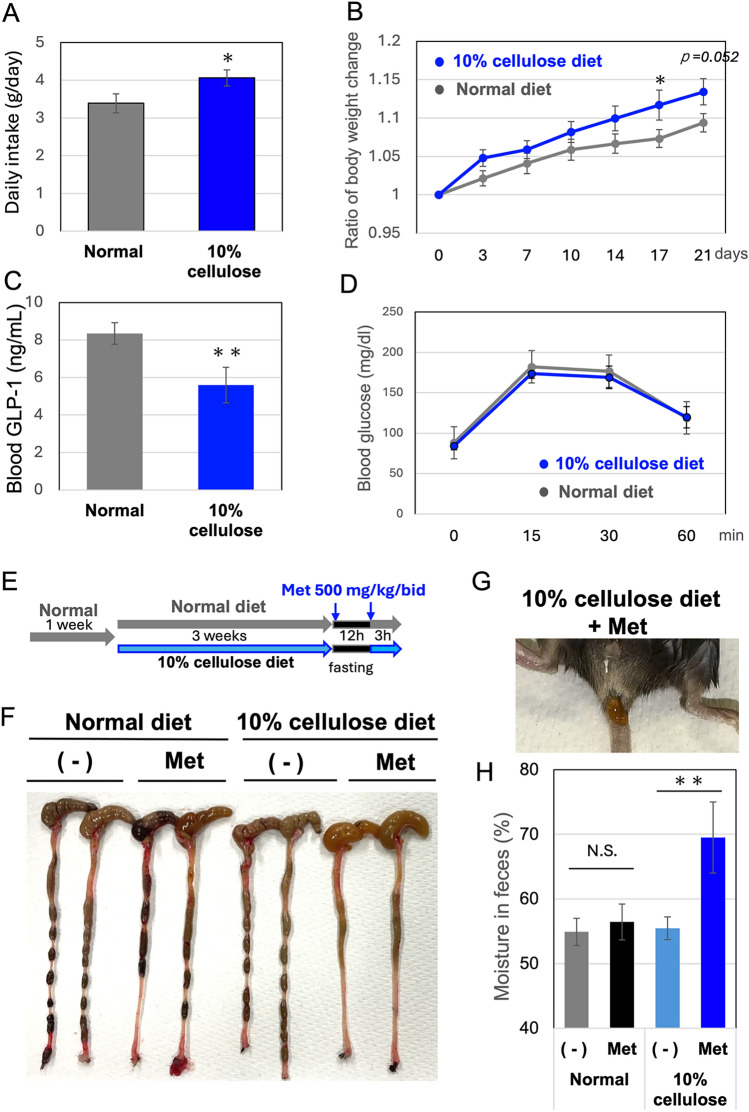
10% cellulose diet and overeating increase fecal water content after metformin treatment **A** Six-week-old male C57BL/6 J mice [Each group consisted of 4 cages (*n* = *4* per cage, total 16 mice)] were fed a normal diet for 1 week. Subsequently, one group continued the normal diet, while the other groups were switched to a 10% cellulose diet for three weeks. Daily intake was monitored every 3 or 4 days from day 7 to 21. B. Bodyweight change monitored. *n* = *16*. * *p* < *0.05*. (*p* = *0.052* at day 21.) At day 21, half of mice (*n* = *8*) were subjected to blood collection for total GLP-1 measurement by ELISA (**C**) followed by a glucose tolerance test performed by orally administering 1 g of glucose (**D**). The remaining mouse groups (*n* = *8* each) were subjected to metformin assays. E. The experimental scheme is shown. Metformin (Met: 500 mg/kg/bid, twice a day) was orally administered before and after fasting (12 h during the night). (-) means without metformin (only water). Images of mice colon (**F**) and feces (**G**) after metformin treatment are shown. H. Water content in mice feces is shown. Means and S.D. are shown. N.S., not significant. ** *p* < *0.01*

To examine whether the 10% cellulose-containing diet increased appetite, which could potentially lead to overeating even in healthy mice, the number of mice was increased, and food intake was monitored. A more than 20% increase (Fig. 2A), followed by enhanced weight gain (Fig. 2B), might be attributed to lowered GLP-1 levels (Fig. 2C), although no significant differences in glucose tolerance were observed (Fig. 2D). These results suggest that overeating is a risk factor for metformin-induced diarrhea in mice, which may be reproduced even in mice that have not developed diabetes or severe obesity. Clear differences in colon appearance and fecal moisture were observed when mice that had been fed the 10% cellulose diet were treated with metformin (Fig. 2E–H). Moreover, the mice adapted to a 10% cellulose diet consumed approximately 1.5 times more food after fasting compared to the normal diet group (Fig. S3), suggesting that overeating may be a trigger of metformin-induced diarrhea.

**Fig. 3:**
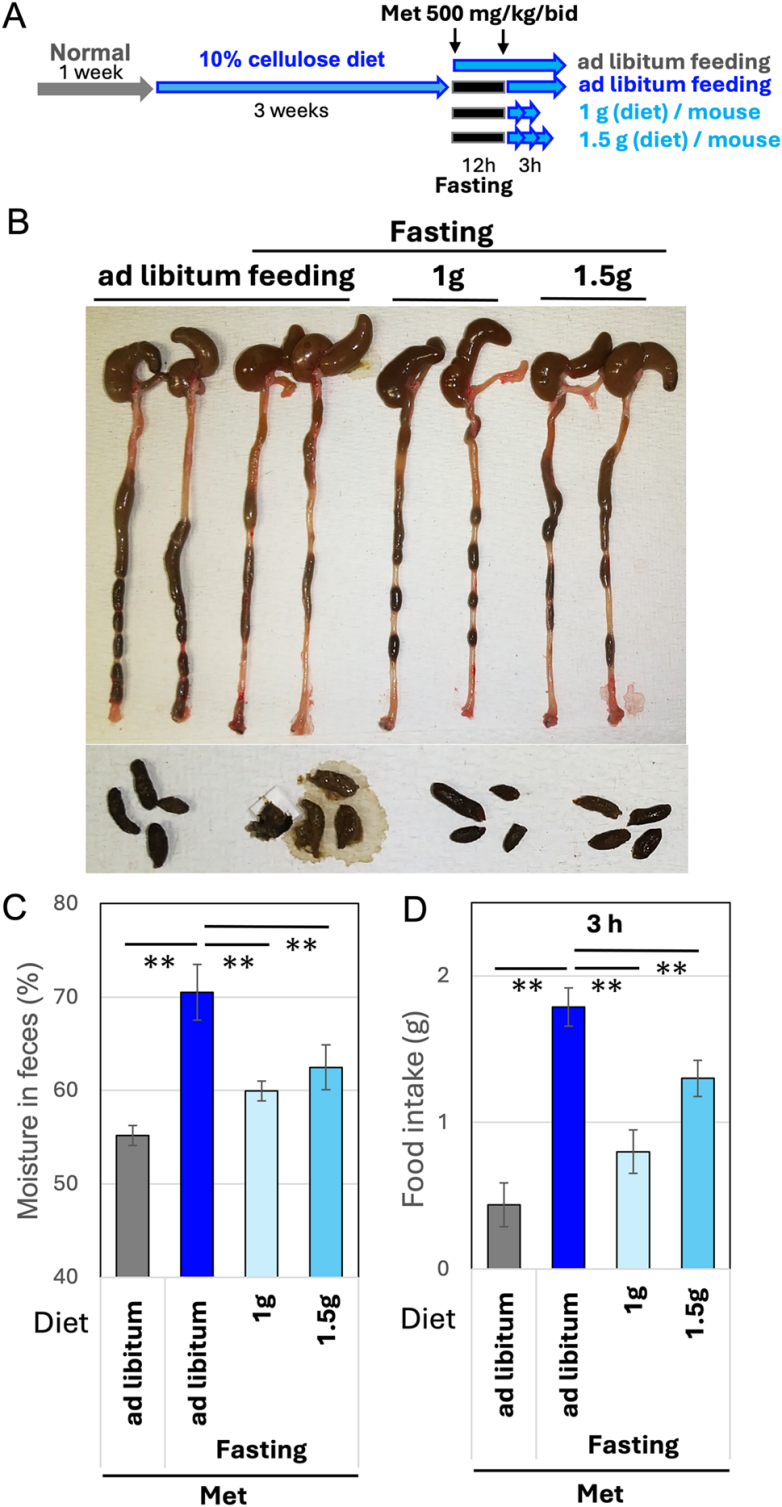
Dietary restriction prevents metformin-induced diarrhea. **A**. Seven-week-old male mice (*n* = *4/*group) were fed a 10% cellulose diet for three weeks and treated with metformin (Met: 500 mg/kg) administered orally (P.O.). One group was allowed ad libitum feeding, while another group was subjected to 12-h nighttime fasting. The fasting groups were further divided into three categories: no dietary restriction or a specified amount of diet per mouse was provided. **B** Images of the colon and feces after 3-h feeding post the 2nd metformin treatment. **C** Fecal moister were measured. Means and S.D. are shown. ** *p* < *0.01*. D. Amount of food actually consumed, excluding spills, during the 3-h feeding post the 2nd metformin treatment

To conclude that overeating is a risk factor in metformin-induced diarrhea, the mice were provided with a larger food intake after the second administration of metformin compared to the conditions shown in Fig. S2. The supplementation of even 1.5 g of diet was insufficient to induce diarrhea caused by metformin (Fig. 3A and B), which was also evaluated based on fecal moisture content (Fig. 3C). Considering that the mice did not consume all the supplied diet (Fig. 3D), at least less than 1.4 g of diet, which is almost the same amount as that consumed by mice fed with a normal diet (Fig. S3), may be insufficient to induce diarrhea.

Next, we examined the involvement of indigestible cellulose and the volume of daily food intake in metformin-induced diarrhea. Since mice consume approximately 4 g/day of the 10% cellulose diet (Fig. 2A), the daily supply of the 10% cellulose diet was limited to 3.5 g/day during the final week for one group. After a 12-h fasting period with metformin administration, the mice were given ad libitum access to either a normal diet or the 10% cellulose diet (Fig. S4A). Mice that had been fed the 10% cellulose diet for 3 weeks under ad libitum conditions exhibited diarrhea-like feces (Fig. S4B) and increased fecal moisture content (Fig. S4C), regardless of the diet type provided during the 3-h refeeding period following the 12-h fast with metformin. Under restricted feeding conditions during the final week, the mice consumed approximately 1.6 g of diet during the 3-h refeeding period (Fig. S4D). These findings suggest that the level of dietary intake, possibly reflecting habitual feeding behavior, may also contribute to metformin-induced diarrhea. However, the absolute amount of food consumed over a short period appears to be the more critical factor.

### Wood creosote alleviates metformin-induced diarrhea independent of the expression of endocrine markers

**Fig. 4:**
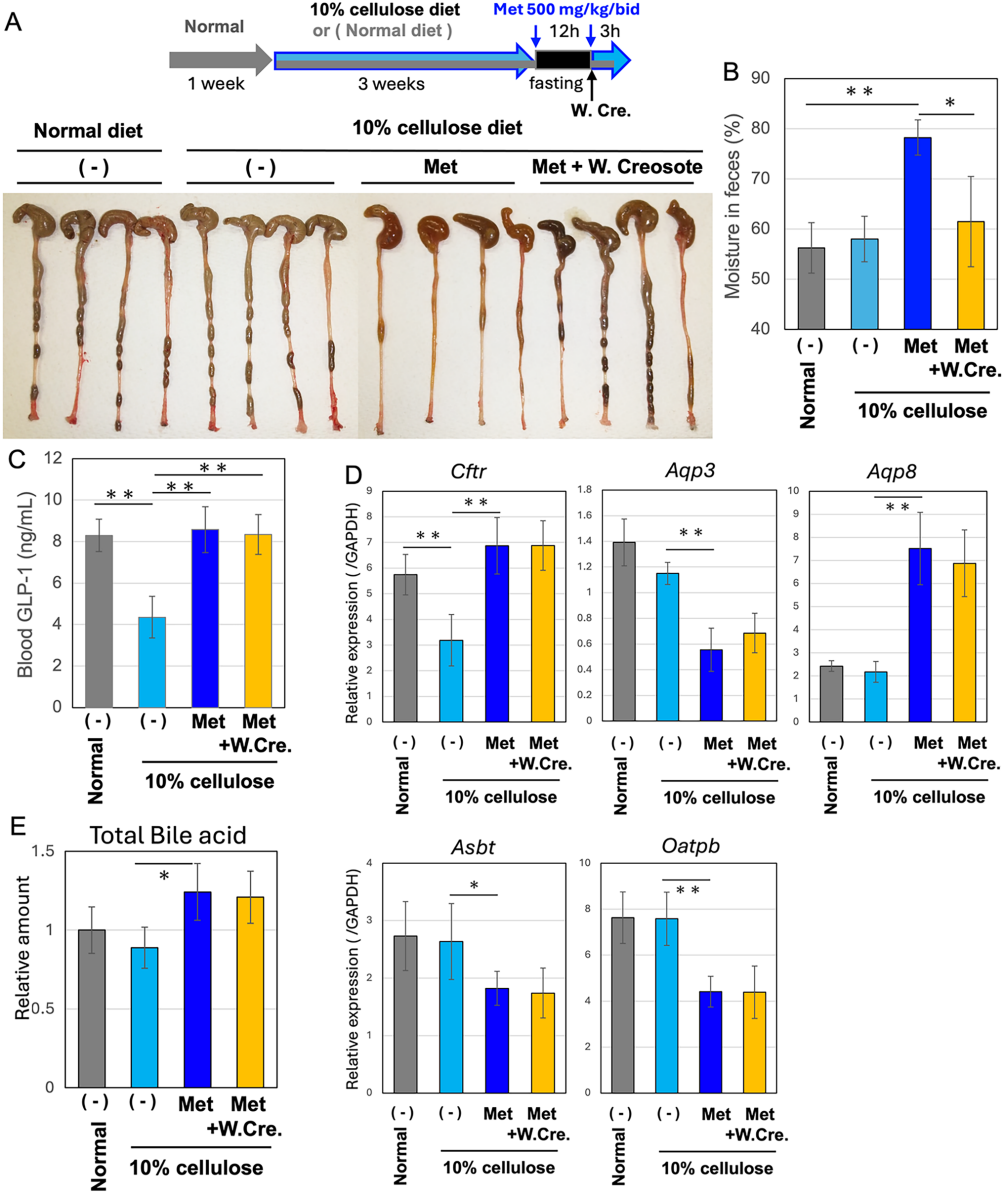
Wood creosote ameliorates diarrhea induced by metformin **A** Seven-week-old male mice that had been fed normal or 10% cellulose diets for three weeks were treated with or without metformin (Met: 500 mg/kg/bid) and wood creosote (5 mg/kg). (-) means without metformin (only water). An image of the colon is shown. Facial moisture (**B**) and blood GLP-1 levels (**C**) were measured. Gray bars indicate the mouse group fed a normal diet and subjected to 12 h fasting [without metformin, (-) in A]. Sky blue bars indicate the mouse group fed a 10% cellulose diet and subjected to 12 h fasting [without metformin, (-) in A]. Dark blue bars indicate the mouse group fed a 10% cellulose diet and subjected to metformin treatment before and after 12 h fasting [Met in A]. Yellow bars indicate the mouse group that was fed a 10% cellulose diet, treated with metformin before and after 12 h of fasting, and administered wood creosote alongside the second metformin treatment [Met + W. Creosote in A]. *n* = *8*. Means and S.D. are shown. * and ** indicate *p* < *0.05* and *p* < *0.01*, respectively. D. mRNA expression was examined by quantitative PCR analyses. The expression levels are shown as relative levels normalized by *Gapdh* mRNA level.* n* = *6*. E. Total bile acid in the ileum was measured using the bile acid quantification kit

Science wood creosote is a candidate for a therapeutic material to treat metformin-induced diarrhea *in db/db mice*, which is also tested in the present overeating model (Fig. 4A). Fecal moisture in mice treated with metformin was significantly recovered by the co-administration of wood creosote (Fig. 4B). Suppressed blood GLP-1 levels increased two-fold with the administration of metformin, but the levels did not vary with wood creosote (Fig. 4C). These results suggested that the increase in GLP-1 secretion in the gut might be a cause of metformin-induced diarrhea, but the mechanism by which wood creosote ameliorate the diarrhea symptom might be differ from the overeating model.

The markers associated with metformin-induced diarrhea in a mouse model of obesity and type 2 diabetes (*db/db*) [7] were also tested in the present overeating model. The mRNA expression of the chloride channel *Cftr* in the ileum was increased by metformin (Fig. 4D), suggesting that, as in *db/db*, an increase in luminal osmolarity is also a cause of diarrhea in the overeating model. Although both *Cftr* and the water channel aquaporin 3 (*Aqp3*) has been reported induced by cAMP/CREB signaling, the *Aqp3* mRNA levels was decreased by metformin. In contrast, the *Aqp8* mRNA level was increased by metformin. The bile acid accumulation in the ileum (Fig. 4E) can be explained by suppression of mRNA expression of two genes,* Asbt* and *Oatpb*, involved in bile acid reuptake.

The treatment with wood creosote did not affect the mRNA expression patterns modified by metformin. These results lead to a speculation that the recovery or amplification rate in GLP-1 expression may be a primary cause of metformin-induced diarrhea and that the combination of dysregulation in several factors involved in osmolality and water balance may impair water absorption in the gut. However, wood creosote may improve water absorption in the colon without affecting the expression of metformin-associated genes, which was also observed in *db/db* mice.

### Loperamide decreases the amount of digestate entering the cecum

Loperamide, a μ-receptor agonist, suppresses acetylcholine secretion in the parasympathetic peripheral neurons, thereby decreasing peristalsis in the digestive tract and relieving diarrhea symptoms [24]. By comparing the actions of wood creosote and loperamide in relieving diarrhea symptoms, the commonalities and differences between the two agents were examined. Like wood creosote, loperamide ameliorates diarrhea symptoms in mice subjected to metformin-induced diarrhea in an overeating model (Fig. S5A). Water content in feces was reduced by loperamide treatment (Fig. S5B). Interestingly, the influx of digestate into the cecum was reduced in the loperamide group (Fig. S5C), while no difference in digestate influx was observed in the wood creosote group. These results suggest that both wood creosote and loperamide could ameliorate diarrhea symptoms induced by the combination of overeating and metformin, but the precise mechanisms by which these drugs reduce water content in feces may not be identical.

### The GLP-1 receptor antagonist Exendin-3 (9–39) prevents diarrhea induced by metformin

**Fig. 5:**
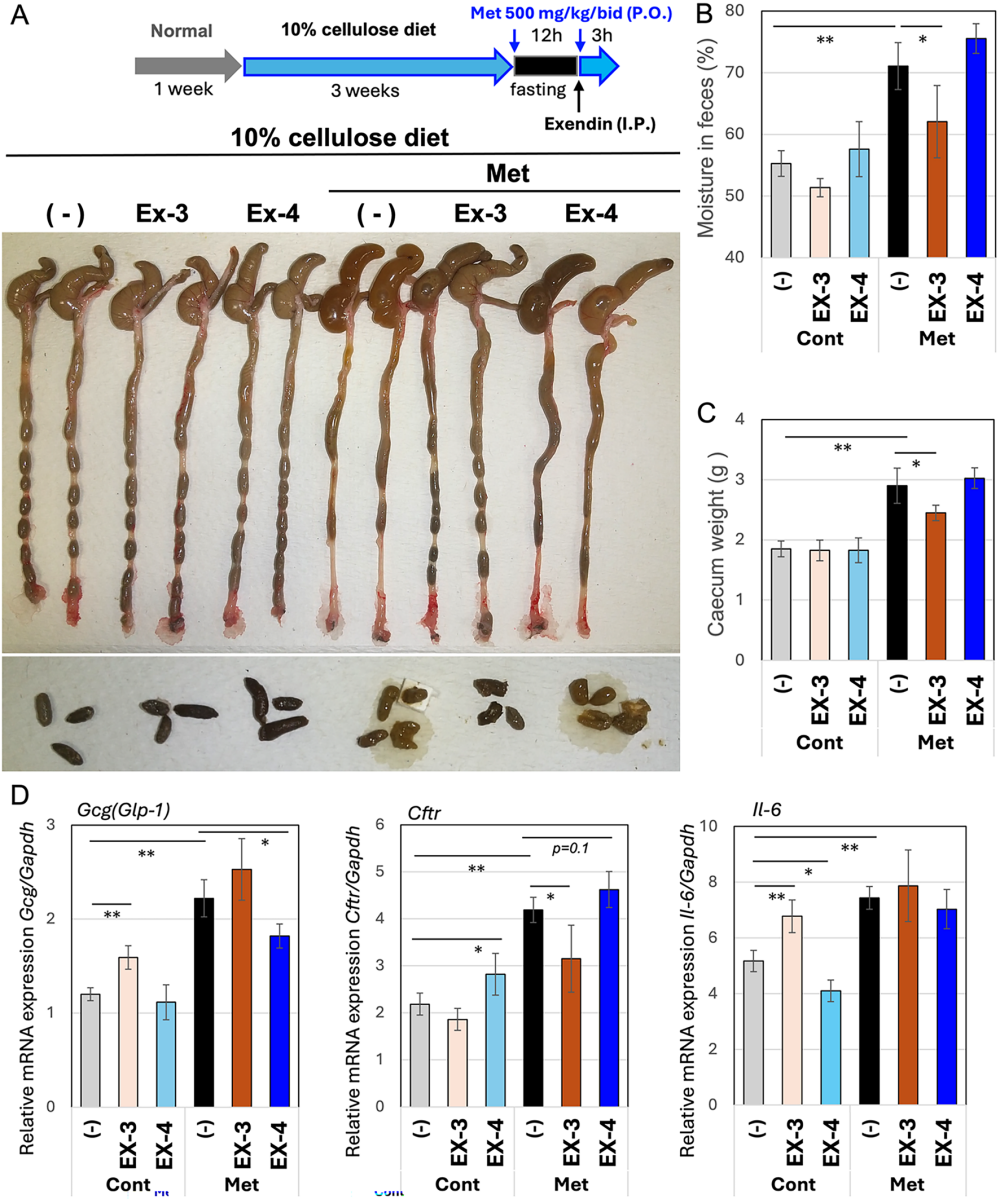
Exendin-3 (9–39) prevents diarrhea induced by metformin **A** Seven-week-old male mice (*n* = *4*) fed either 10% cellulose diet for three weeks were treated with metformin (Met: 500 mg/kg) administered orally (P.O.). Following a 12-h fasting period, the mice received Exendin-3 (9-39) or Exendin-4 (1 μg each in PBS) via intraperitoneal administration (I.P.). Then, the mice were administered metformin (500 mg/kg). After 3 h of re-feeding, the colon was collected (upper), and feces were recovered (lower). (-) means without GLP-1 receptor agonist/antagonist (only PBS). Facial moisture (**B**) and cecum weight (**C**) were measured. Means and S.D. are presented, with * and ** indicating *p* < *0.05* and *p* < *0.01*, respectively. **D** Total RNA was extracted from the ileum, and mRNA levels of Glucagon [*Gcg* (Glp-1)], *Cftr*, and *Il-6* were quantified by qPCR, normalized to *Gapdh* mRNA levels

We investigated the role of GLP-1 in metformin-induced diarrhea, as increased GLP-1 levels have been linked to diarrhea symptoms not only in this study but also in our previous study using *db/db* mice [8]. The mice were fed a 10% cellulose diet for three weeks and then intraperitoneally administered the GLP-1 receptor antagonist Exendin-3 (9–39) prior to the second metformin administration after 12 h fasting. While Exendin-3 (9–39) showed minimal or no effects on the condition of the colon or feces (including fecal moisture) in mice without metformin treatment, it effectively prevented diarrhea-like symptoms in mice treated with metformin (Fig. 5A and 5B). The cecum weight was also reduced by Exendin-3 (9–39) in mice treated with metformin (Fig. 5C). In contrast, the administration of the GLP-1 receptor agonist Exendin-4 was insufficient to induce diarrhea in mice without metformin, although there was a tendency for increased fecal moisture in mice treated with metformin.

Diarrhea induced by metformin in *db/db* mice is associated with increased mRNA expression of Glucagon (*Gcg*, which encodes GLP-1) and the chloride channel *Cftr* [8]. The potent biological effects of Exendin-3 and Exendin-4 were evaluated based on changes in these mRNA levels (Fig. 5D). Notably, the reduction of diarrhea symptoms was accompanied by a decrease in *Cftr* mRNA expression in metformin treated mice. Since the pro-inflammatory cytokine IL-6 and its signaling have been reported to be associated with GLP-1 signaling and metformin signaling [21, 25, 26], the mRNA levels of *Il-6* were also examined. Exendin-3 (9–39) and Exendin-4 modulated *Il-6* mRNA levels up and down, respectively, in metformin-untreated mice. In contrast, neither Exendin-3 nor Exendin-4 affected *Il-6* mRNA levels in metformin-treated mice, despite the enhanced expression induced by metformin. These results suggest that inflammation may not be linked to metformin-induced diarrhea.

To compare the present overeating model with *db/db* mice [7], fecal microbes were also examined. Similar to metformin-induced diarrhea in *db/db* mice, the population of the *Firmicutes* family in feces was reduced when overeating mice exhibited diarrhea (Fig. S6A and B). These results suggest that, in the context of a metformin-induced diarrhea model, the present transient overeating model may resemble the genetic overeating mouse model (*db/db*).

## Discussion

In this study, we found that metformin causes diarrhea-like symptoms in healthy mice, when accompanied by abnormal feeding behavior. The abnormal feeding behavior may be related to the amount of food consumed within a specific time period.

To induce diarrhea, the amount of diet may need to exceed 1.4 g, which corresponds to approximately 50%–60% of the daily intake of 10-week-old male C57BL/6 J mice. Additionally, it is important to note that pretreatment with 500 mg/kg of metformin prior to a fasting period of 12 h, followed by a secondary administration of the same dose of metformin, is necessary to induce diarrhea. These results suggest that while metformin induces diarrhea, it primarily acts to increase the risk of developing diarrhea as a consequence of overeating.

In general, type 2 diabetes is caused by excessive calorie intake followed by weight gain [27]. Therefore, individuals with type 2 diabetes are often accompanied by obesity. Feeding behavior is proposed to be a risk factor of obesity, type 2 diabetes, and diarrhea [28, 29]. Binge eating disorder is psychological disorder associated with gastrointestinal symptoms, such as heartburn and diarrhea. Unlike humans, the mice can only reproduce overeating in certain genetic backgrounds, such as *db/db* [24]. Unexpectedly, a low-calorie diet containing 10% cellulose resulted in overeating and weight gain in mice. The 10% cellulose diet contains fewer calories per volume compared to the normal diet, leading to increased appetite due to reduced GLP-1 circulation. Conversely, the fibers (cellulose) in the diet inhibit glucose absorption in the gut, potentially enhancing GLP-1 expression in the ileum [30]. Thus, the effects of acute and chronic ingestion of fiber-containing diets on GLP-1 expression, along with appetite regulation, warrant further investigation.

Despite no difference in glucose tolerance, the results after consumption of caloric restricted diets were contrary to the typical impression of meals suitable for individuals with diabetes. However, it is true that Japanese consume a diet higher in dietary fiber than Westerners and are prone to diarrhea if they prescribed metformin after developing type 2 diabetes [6]. C57BL/6 J mice did not develop metformin-induced diarrhea after ingestion of a 10% cellulose diet alone. These results suggest that the behavior of feeding all at once over a short period of time is a cause of metformin-induced diarrhea.

Previously, we reported that metformin-induced diarrhea in *db/db* mice was associated with enhanced expression of GLP-1, which induces the Cl^−^ channel CFTR in a diarrhea-associated cAMP cascade [8]. Therefore, in the present study, we monitored GLP-1, CFTR, and other transporters/channels as markers for metformin-induced diarrhea [8]. GLP-1 is secreted from L-cells in the illume and colon after meal. GLP-1 stimulates insulin secretion in pancreatic β-cells, decreases intestinal motility, and suppresses appetite [31–34]. GLP-1 and its receptor agonists are medications of eating disorders, overeating. Although the incidence is not as high as with metformin, diarrhea is an adverse effect of GLP-1 [35, 36]. The difference between metformin and GLP-1 receptor agonists may lie in the amount of food intake, which tends to be higher in individuals prescribed metformin compared to those on GLP-1 receptor agonists.

In the present overeating model, however, the serum GLP-1 levels are decreased in mice which were fed a 10% cellulose-containing diet, which explains the overeating. When the mice were treated with metformin, the GLP-1 levels increased to those observed in the control mice that were fed a normal diet. We speculate here that diarrhea symptoms are not induced by absolute GLP-1 concentrations, but rather by the relative increase from the steady state. Additionally, CFTR expression levels were found to correlate with GLP-1 levels, consistent with a previous report [8]. Experiments with a GLP-1 receptor agonist and antagonist suggested that administration of the GLP-1 agonist was insufficient to induce diarrhea in mice without metformin treatment. However, diarrhea induced by metformin was inhibited by the GLP-1 receptor antagonist, suggesting that GLP-1 receptor signaling may contribute to diarrhea in conjunction with other signaling pathways induced by metformin in mice after overeating.

Daily Japanese meals contain higher cellulose content compared to Western meals. However, some studies have shown that GLP-1 induction rates after meals are lower in the Japanese population [37]. Additionally, the incidence of metformin-induced diarrhea is higher among the Japanese [6]. These reports in Japanese individuals with diabetes may be comparable to the metformin-induced diarrhea model observed in mice fed a cellulose-rich diet.

When the present diarrhea model mice were treated with approved medications, wood creosote or loperamide, both treatments effectively relieved diarrhea symptoms. Since these were not accompanied by changes in serum GLP-1 levels, it was suggested that the relief of diarrhea by these drugs does not affect the treatment of type 2 diabetes. Moreover, the results of experiments using wood creosote or loperamide suggest that multiple factors, in addition to GLP-1 signaling, contribute to the development of metformin-induced diarrhea.

Growth-differentiation factor-15 (GDF15), a peptide hormone belonging to the transforming growth factor β superfamily, has been shown to reduce food intake and lower body weight [38]. GDF15 is expressed in the gastrointestinal tract, kidney, liver, and adipose tissue, and is induced by metformin treatment in humans and mice (in mice, a single administration of 600 mg/kg increases serum GDF15 levels by sixfold). Pair-fed, a method used to adjust food intake to the same level, also demonstrated that GDF15 promotes calorie consumption and induces weight loss [39]. Since the action of GDF15 is mediated through its receptor, GDNF family receptor α-like, in the area postrema and the nucleus tractus solitarii of the brainstem, it does not directly regulate the gastrointestinal tract. GDF15 is also a mediator of nausea and emesis induced by agents such as the anticancer drug cisplatin, which is expected to be associated with serotonin-mediated diarrhea. However, the administration of the serotonin receptor antagonist ondansetron to rodents suppressed anorexia but did not affect alternative behavioral indicators of nausea [40].

Mitochondrial dysfunction reduces serum GDF15 levels, while imeglimin, an anti-diabetic drug structurally related to metformin, improves mitochondrial function and increases GDF15 levels [41]. A major adverse effect of imeglimin is gastrointestinal symptoms, including diarrhea (observed in nearly 40% of individuals prescribed imeglimin) [42], suggesting that metformin-induced diarrhea may result from a combination of GLP-1 and GDF15.

In speculation, metformin-induced diarrhea in model mice is a type of diarrhea resulting from abnormal feeding behavior occasionally experienced in our daily life. Metformin prescription is suggested to be one of the factors that increase the probability of its incidence. In addition to feeding behavior, a high-fiber diet for type 2 diabetes may also increase the risk of metformin-induced diarrhea.

## Supplementary Information

Below is the link to the electronic supplementary material.

Supplementary file1 (PDF 6342 KB)

## Data Availability

The data that support the findings of this study are available from the corresponding author upon reasonable request.

## Declarations

### Animal research

For studies with animals, the care and use of experimental animals were conducted in accordance with all institutional and national guidelines. In this study, we did not use human materials.
