# Supplementary material for: Metformin induces diarrhea in mice under over-eating conditions
Source: Diabetol Int. 2025 Jun 5;16(3):568–79. doi: 10.1007/s13340-025-00822-0 (PMC12209130; doi:10.1007/s13340-025-00822-0)
Supplement: Supplementary file 1 — Supplementary file1 (PDF 6342 KB) [file 13340_2025_822_MOESM1_ESM.pdf]

Supplementary Table

Gene Symbol	Species	Sequence
Gcg (*Glp-1*)	mouse	5'-GTGCAGTGGTTGATGAACAC
		5'-GCCTTTCACCAGCCAAGCAA
Cftr	mouse	5'-CTTGCAAGTGGCACTCCTCA
		5'-CTGAGGTAATCACAAGTCTTTCACT
Aqp3	mouse	5'-CTGGACACTTGGACATGGTCA
		5'-CATGGAGGTTCCAATGACCA
Aqp8	mouse	5'-GGCTAAAGTGGTGAGTCCAGA
		5'-CAGCACCCATACACACAGCCAA
Asbt	mouse	5’- CTACACCAAGATGTGGGTTGACT
		5’- GGGTTCAATGATCCAGGCACT
Oatpb	mouse	5’- CGACTTTGCCCACCATAGCAA
		5’- GACCACCAGTCCTGAAGAGAT
Gaphf	mouse	5'-ACTCACGGCAAATTCAACGG
		5'-GACTCCACGACATACTGAGC
Il-6	mouse	5'- GCAATGGCAATTCTGATTGTAT
		5'-GAAGGACTCTGGCTTTGTCTTT
Universal	Entero bacteria	5'- AAACTCAAAKGAATTGACGG
		5'-CTCACRRCACGAGCTGAC
Firmicutes	Entero bacteria	5'- GGAGYATGTGGTTTAATTCGAAGCA
		5'-AGCTGACGACAACCATGCAC
Betaproteo bacteria	Entero bacteria	5'- AACGCGAAAAACCTTACCTACC
		5'-TGCCCTTTCGTAGCAACTAGTG
Bacteroidetes	Entero bacteria	5'- GTTTAATTCGATGATACGCGAG
		5'-TTAASCCGACACCTCACGG

Supplementary Figure 1

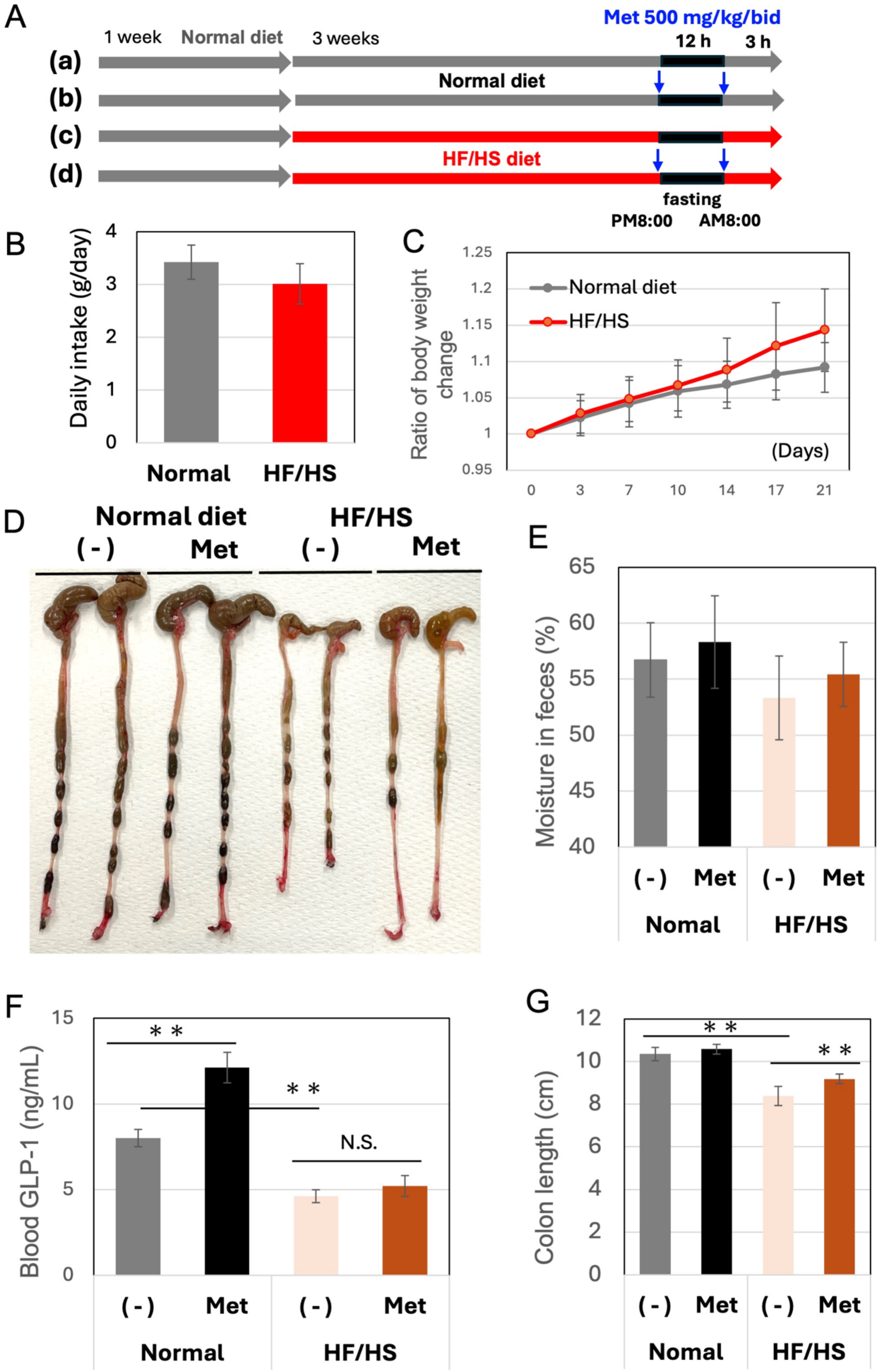

Fig. S1 A. The experimental scheme is shown. Seven-week-old male C57BL/6J mice were fed a high-fat/high-sucrose (HF/HS) diet for three weeks. Metformin (Met: 500 mg/kg, twice a day) was orally administered before and after a 12-h nighttime fasting period. Daily food intake (B) and body weight changes (C) were monitored (*n=8*). Images of the mouse colon (D) and water content (E) in feces are shown. Means and standard deviations (S.D.) are shown. Blood GLP-1 levels (F) and colon length (G) were measured. $**p < 0.01$. N.S. not significant.

Supplementary Figure 2

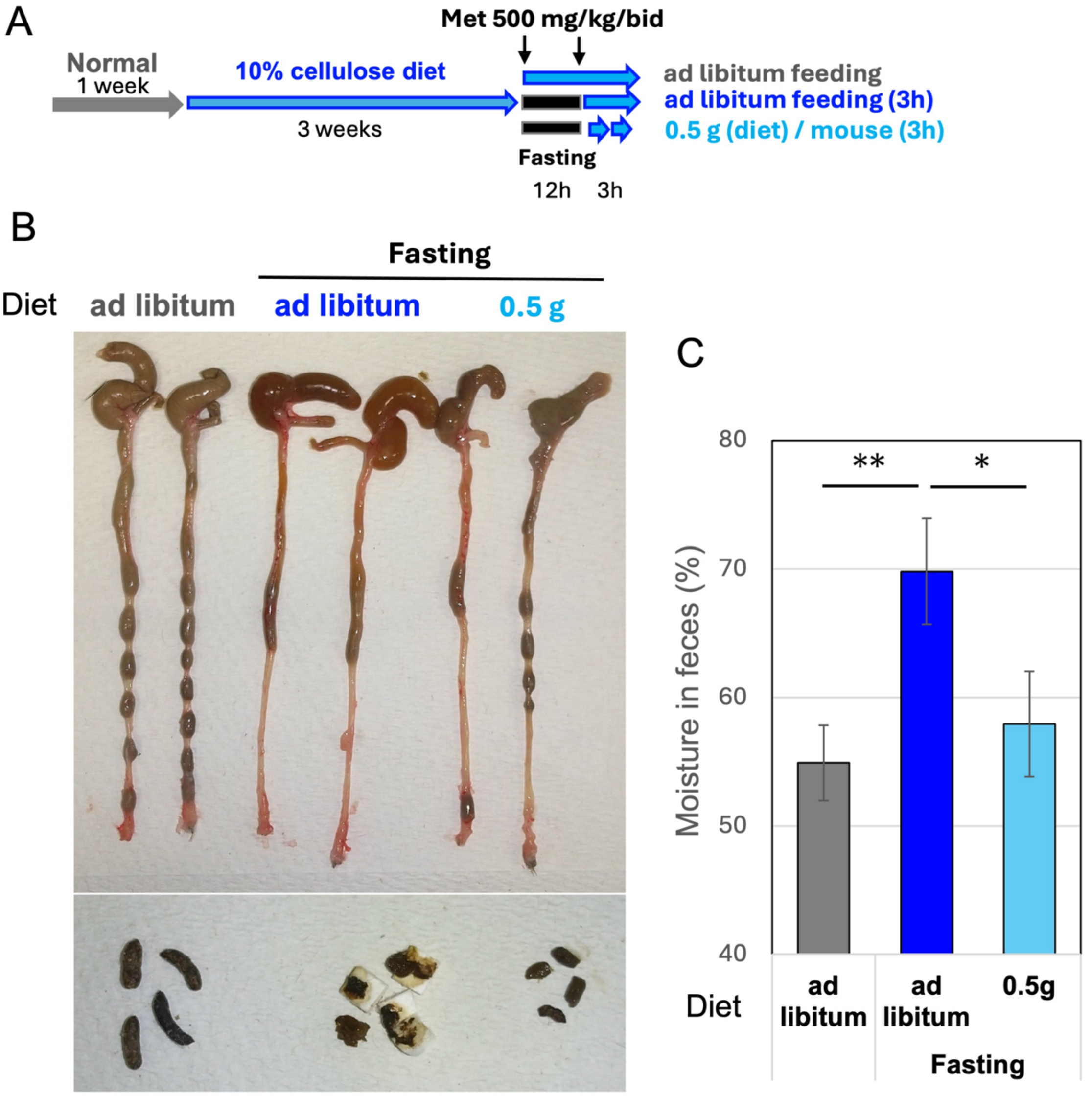

Fig. S2 A. The experimental scheme is shown. Seven-week-old male C57BL/6J mice (*n=4*) were fed a 10% cellulose diet for three weeks. Metformin (Met: 500 mg/kg, twice a day) was orally administered without dietary restriction [Group: *ad libitum*]. Metformin was administered before and after a 12-h nighttime fasting period. After fasting, the mice were placed under conditions without dietary restriction for 3 h [Group: *ad libitum* (3h)]. After the second dose of metformin, 0.5 g of diet (crushed pellets) was provided to the mice [Group: 0.5 g (diet)/mouse (3h)]. B. Images of the Colon (top) and Feces (bottom). C. Facial Moisture Measurement. Means and standard deviations (S.D.) are shown. *: $p < 0.05$, **: $p < 0.01$.

Supplementary Figure 3

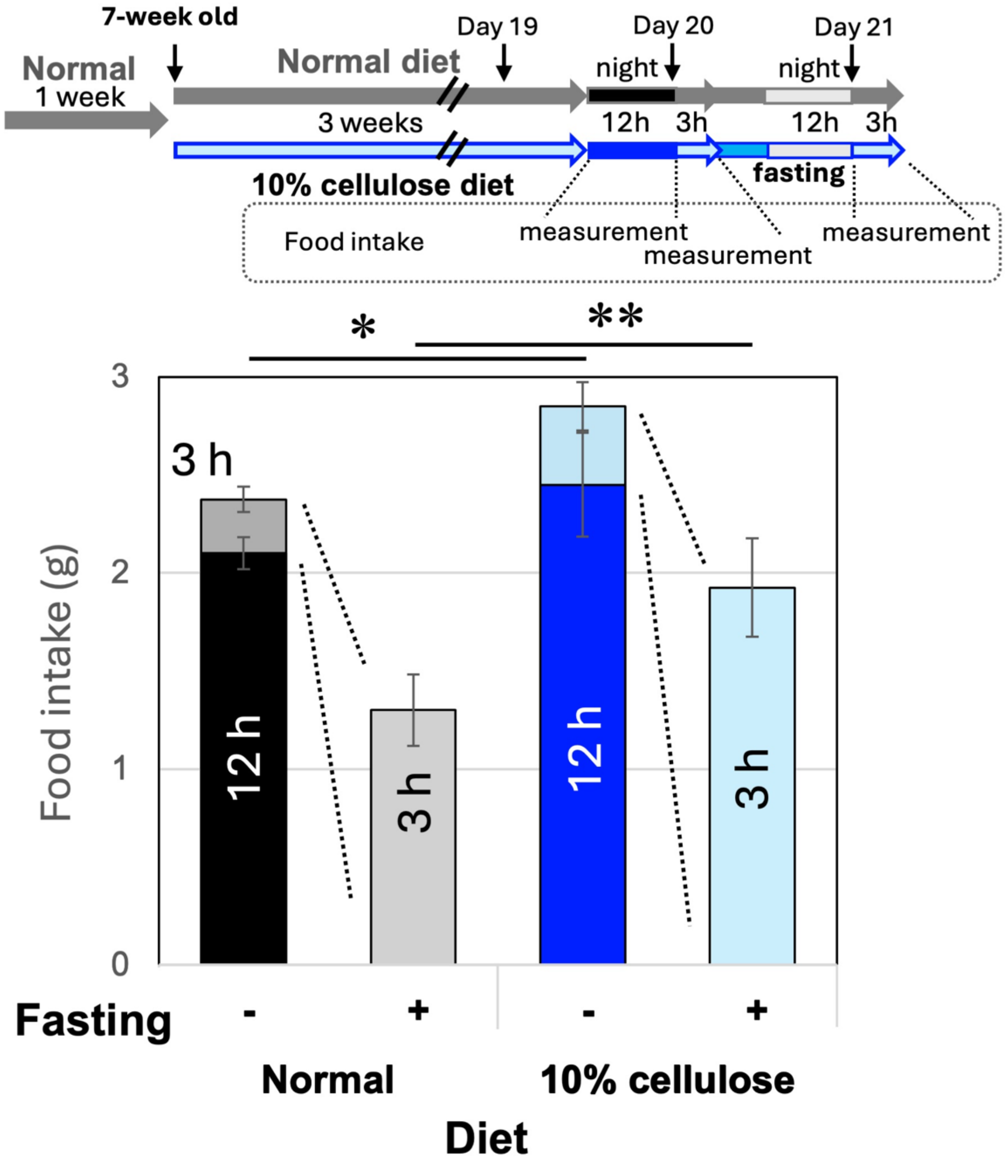

Fig. S3 Seven-week-old male C57BL/6J mice ($n = 4$) were fed either a normal diet or a 10% cellulose diet for three weeks. Food consumption was measured during a 12-h nocturnal period (night of day 19) and a 3-h morning period (day 20). The mice were subsequently fasted for 12 h overnight, and their food intake was measured over a 3-h period the following morning (day 21). (Fasting - : day 19~20. Fasting + : day 21.) Data are presented as means and S.D. *: $p < 0.05$, **: $p < 0.01$.

Supplementary Figure 4

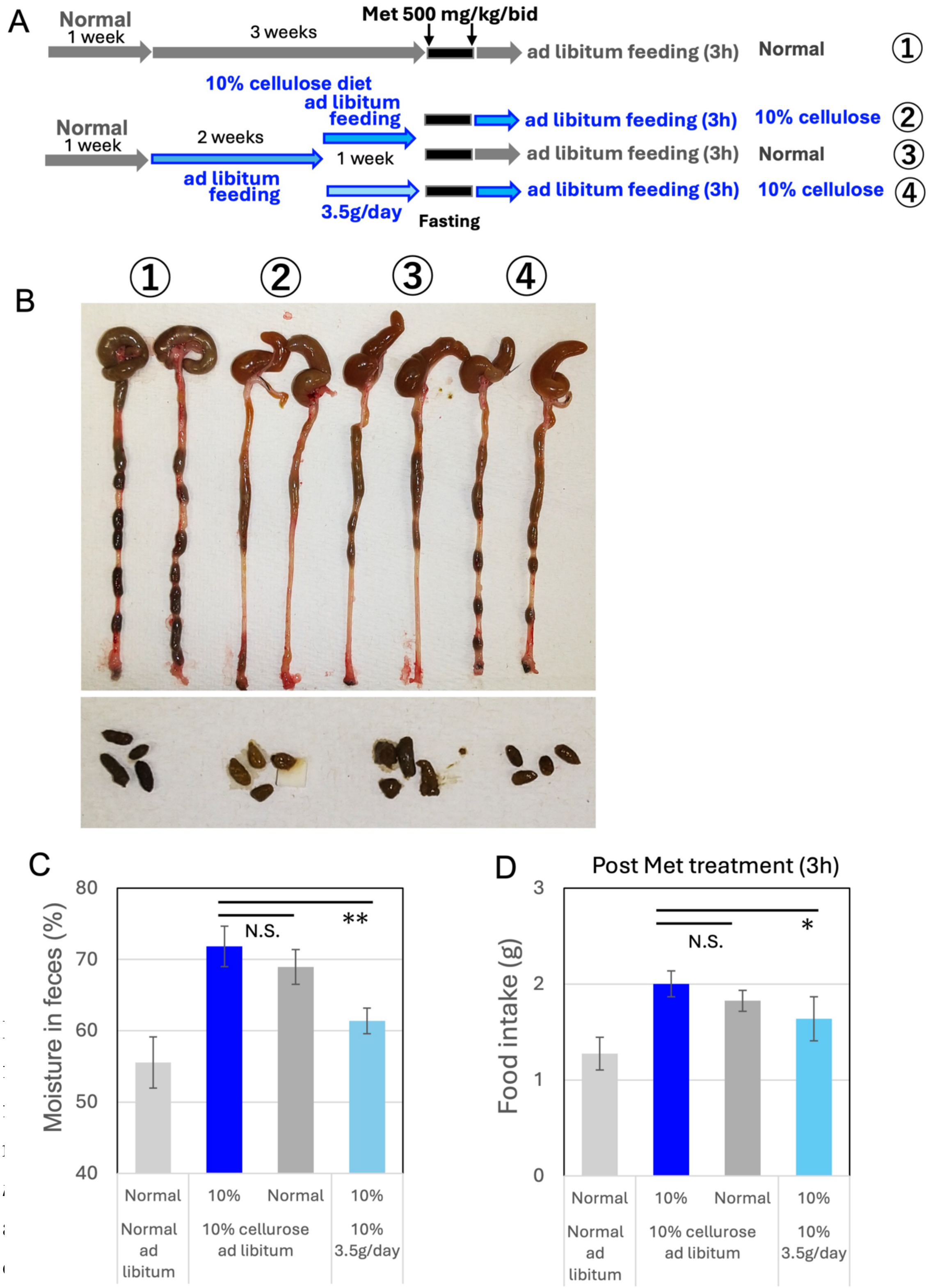

Fig. S4 A. Seven-week-old male C57BL/6J mice (*n* = *4*) were fed either a normal diet or a 10% cellulose diet for three weeks. The group receiving the 10% cellulose diet was further divided into two subgroups: one fed *ad libitum* and the other subjected to a restricted diet (3.5g/day) during the final week. Metformin (Met; 500 mg/kg, twice daily) was orally administered before and after a 12-h overnight fasting period. Following the fasting period, the mice were allowed *ad libitum* (3 h) access to either a normal diet or a 10% cellulose diet. B. Images of the Colon (*upper*) and Feces (*lower*). C. Facial Moisture Measurement. D. The quantity of diet consumed by mice within the final 3 h. Means and S.D. are shown. *: $p < 0.05$, **: $p < 0.01$.

Supplementary Figure 5

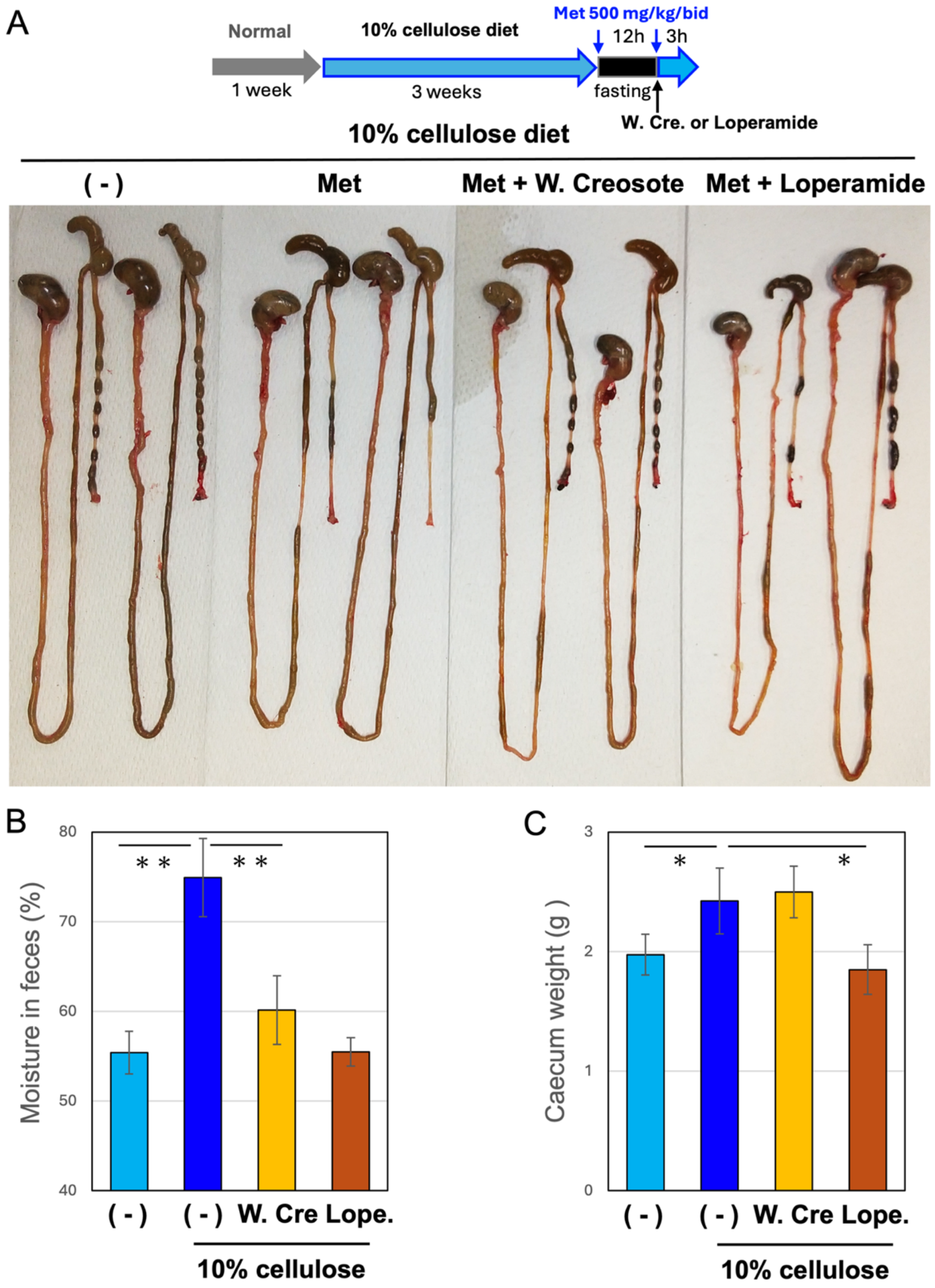

Fig. S5 A. Seven-week-old male mice that had been fed normal or 10% cellulose diets for three weeks were treated with metformin (Met: 500 mg/kg/bid) and wood creosote (5 mg/kg/bid) or loperamide (10 mg/kg/bid). (-) means without metformin (only water). An image of the gut is shown. Facial moisture (B) and caecum weight (C) were measured. $n = 8$. Means and S.D. are shown. * and ** indicate $p < 0.05$ and $p < 0.01$, respectively.

Supplementary Figure 6

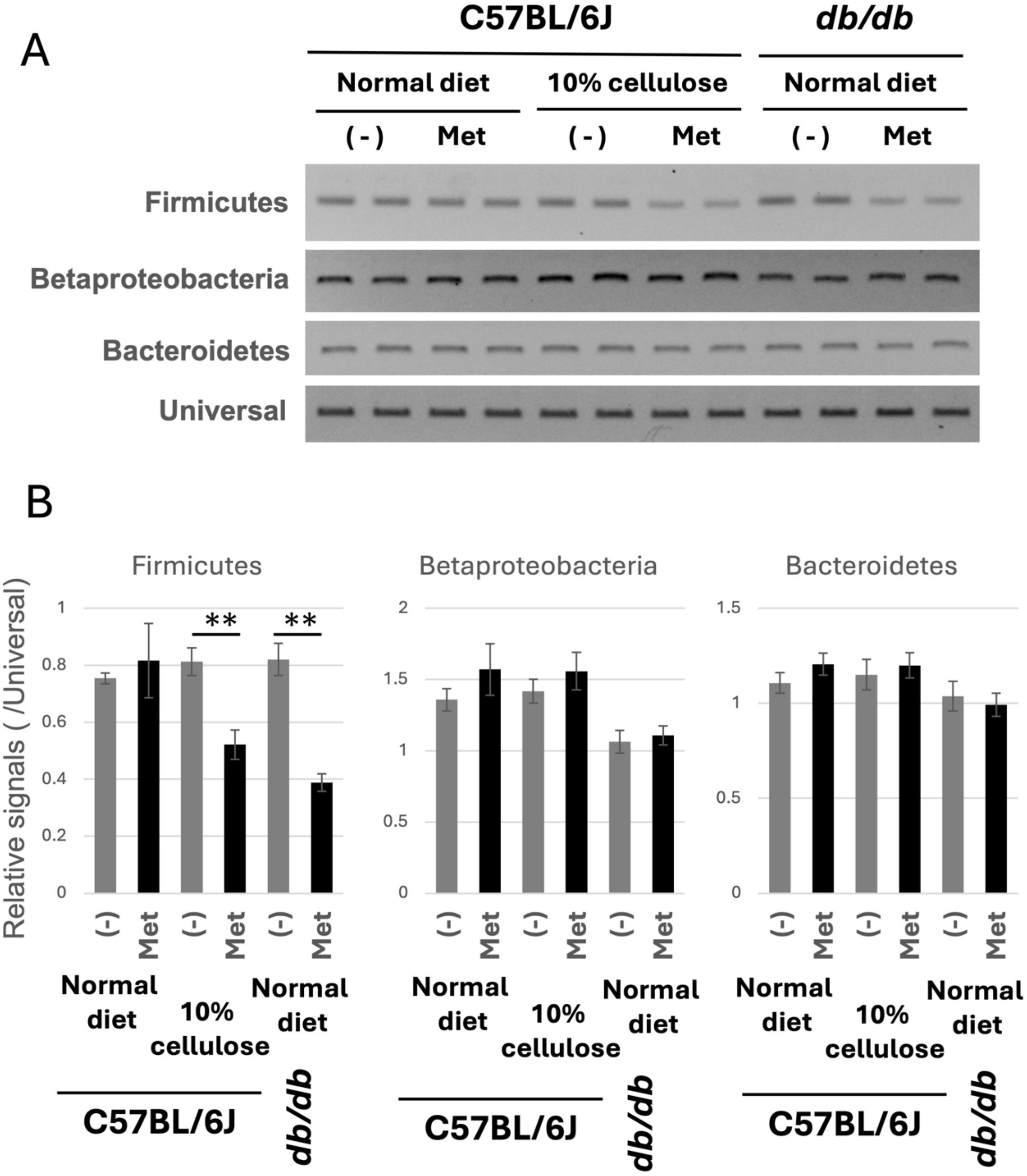

Fig. S6 A. Mice were fed either a normal diet or a diet supplemented with 10% cellulose for 3 weeks. Fecal samples were then collected after a 3-h period following the second administration of metformin. To assess changes in the intestinal microbiota, total DNA was extracted from the feces and analyzed by qPCR as described in [7]. For comparison, DNA samples preserved in the study [7] were used. Briefly, DNA was extracted from fecal samples of db/db mice treated with metformin (500 mg/kg, twice per day for 2 days). Representative agarose gel images from two sets after 30 cycles of PCR are shown. B. qPCR signals for representative bacterial species were normalized to the signals obtained with a universal primer set. *n = 4*. Mean and S.D. are shown. $p < 0.01$.
